# Perceptions, Disease Representations, and Response Obstacles Regarding the Ebola Virus Disease Epidemic in the North Kivu and Ituri Provinces of the Democratic Republic of the Congo

**DOI:** 10.29245/2578-3009/2023/S3.1112

**Published:** 2023-05-12

**Authors:** Nkechi Onyeneho, Joseph Okeibunor, Ijeoma Igwe, Ngozi Idemili Aronu, Amadou Baïlo DIALLO, Tieman Diarra, Barry Rodrigue, Michel N’da Konan Yao, Mamoudou Harouna Djingarey, Ibrahima Socé FALL

**Affiliations:** 1 University of Nigeria, Nsukka; 2 World Health Organization; 3 Independent Consultant, Mali; 4 Independent Public Health Expert, Niger

**Keywords:** Perceptions, Representations, Etiology, Traditional medicine, Modern medicine

## Abstract

We explored the perceptions and representations of diseases in the North Kivu and Ituri provinces of the Democratic Republic of Congo to identify perceived obstacles regarding responses to the country ‘s Ebola virus disease (EVD) outbreak using a mix-methods approach. We surveyed a representative sample including 800 adults aged 18 years and older, held in-depth interviews with 17 community leaders, and conducted 10 focus group discussions with community members (using same-sex interviewers/discussion leaders). The results revealed the existence of several health conditions among members of the two communities. Locals consider nearly 80 of these ailments as untreatable by orthodox medicines and methods, even when symptoms are similar to EVD. Creating awareness must be considered a critical goal of community education to further educate these populations about EVD and other health problems and their respective treatments.

## Introduction

Throughout time, humans have grappled with the occurrence of disease. While understanding how diseases manifest is important, knowing how community members perceive diseases is also crucial to providing effective treatment. Representations and perceptions of diseases clarify people’s behaviors regarding a disease, their understanding of its etiology, and their need to explain its origins^1^. An illness is not only a specific incident but also an event whose meaning is sought. Perceptions and representations provide the foundation for this meaning and are expressed through the names assigned to diseases and the meanings that they carry.

Perceptions and representations of illnesses are multifaceted^2^. Beyond the events constituted by diseases, people’s perceptions and representations of them describe their etiology and influence treatment possibilities. If a disease is considered the result of a curse, evil phenomenon, transgression, breaking of a taboo, aggression, the manipulation of evil forces, or as a punishment, the available resources to promote healing and regain well-being must be used for treatment. The etiology of a disease reveals its nature and source, but researchers have demonstrated that, in some cultures, Ebola virus disease (EVD) is often perceived as a devilish or demonic disease or a conjured attack^3, 4^. In communities in the Democratic Republic of the Congo’s North Kivu and Ituri provinces, the names people assign to diseases convey these perceptions and representations with specific meanings.

In this study, which was conducted with community members in North Kivu and Ituri, we focused on identifying and analyzing the perceptions and representations of various diseases mentioned by the study participants and their treatments for those diseases. Specifically, we examined EVD in the context of the North Kivu and Ituri provinces of the Democratic Republic of Congo (DRC) by documenting the experiences and lessons around the communities’ responses to the 10th EVD outbreak.

The study was conducted in North Kivu and Ituri, both of which are provinces that were affected by the 10th EVD outbreak in the DRC. **Ituri**, one of the 26 DRC, has as its capital the city of Bunia, which encompasses the Ituri Rainforest. Located northeast of the Ituri River and on the western side of Lake Albert, Ituri is a region with high plateaus (2000–5000 meters) and a large tropical forest, but it also comprises the landscape of the savannah. The district has rare fauna, including the okapi—the national animal of the Congo. As for flora, an important species is Mangongo, whose leaves are used by the Mbuti to build their homes. The population is composed primarily of Alur, Hema, Lendu, Ngiti, Bira, and Ndo-Okebo peoples, with differing figures as to which of the groups constitutes the largest percentage of the province’s population. The Mbuti, a pygmy ethnic group, reside primarily in the Ituri forest near the Okapi Wildlife Reserve, though some Mbuti have been forced into urban areas by deforestation, overhunting, and violence. Additionally, the Kilo-Moto gold mines are partly located in Ituri. Recent events have brought attention to the Ituri province, as the *Heritage Oil and Gas* and *Tullow Oil* companies found petroleum reserves on the shores of Lake Albert at the beginning of the 21st century.

North Kivu (in French, *Nord-Kivu*) is a province bordering Lake Kivu in the eastern Democratic Republic of the Congo, with Goma as its capital. North Kivu borders the provinces of Ituri to the north, Tshopo to the northwest, Maniema to the southwest, and South Kivu to the south. The countries of Uganda and Rwandalie lie across the eastern border. The province comprises three cities— Goma, Butembo, and Beni—and six territories—Beni, Lubero, Masisi, Rutshuru, Nyiragongo, and Walikale—and is home to the Virunga National Park, a World Heritage Site containing the endangered mountain gorillas. Except for the heightened insecurity and isolation due to rebel activities, North Kivu shares similar demographics with Ituri. However, the North Kivu province is politically unstable and, since 1998, has been one of the flashpoints of the military conflicts in the region.

The “2018 or 10th Kivu EVD outbreak” began on August 1, 2018, when four cases were confirmed, testing positive for the Ebola virus in the eastern region of Kivu in the Democratic Republic of the Congo^5–7^. The Kivu outbreak included Ituri Province shortly after the first EVD case was confirmed on August 13, 2018^8^. Moreover, this outbreak started just days after the end of the 2018 Équateur province Democratic Republic of the Congo EVD outbreak^9–10^. As of the writing of this paper, the affected province and general area are engaged in a military conflict that is hindering EVD treatment and prevention efforts. The World Health Organization’s (WHO’s) Deputy Director-General for Emergency Preparedness and Response has described the combination of military conflict and civilian distress as a potential “perfect storm” that could lead to a rapid worsening of the outbreak^11^. Owing to the deteriorating situation in North Kivu and surrounding areas, the WHO raised the risk assessment at the national and regional level from “high” to “very high” on September 27th^11^.

## Study Design

We adopted a cross-sectional study design with mixed-method data collection techniques. This design allows for multiple windows in which we could harvest data. The use of both quantitative and qualitative approaches to data collection provides the benefits of both while guaranteeing the integrity and robust interpretation and conclusions that the study topic and evaluation deserve.

### Study Population

We sought to include, as the study participants, adults ages ≥18 years living in the community in the study region (the Ituria and North Kivu provinces) and EVD response-team members. A 2010 estimate put the population of North Kivu at 5,767,945. Taking 70% of that number as the 18 years and older population (4,614,356) and an annual growth rate of 3.2%, we estimated the 2019 North Kivu total population as 7,658,406 (all ages) and 5,360,884 (ages ≥18 years). For Ituri, a 2005 estimate put the population at 4,037,561. An estimate of 70% of the population as age ≥18 years = 2,968,865. For 2019, the total population estimate is 6,275,305 (all ages), and the age ≥18 years population is estimated at 4,392,714.

The EVD response team comprised more than 10,000 persons. The team is divided into several different response pillars, including *surveillance, risk communication, social anthropology, vaccination, infection prevention and control, treatment and care, safe and dignified burial, security, logistics*, and *administration*, among others.

### Survey Sample Size Estimation and Sampling strategy Sample size

This was an exploratory study. However, to achieve statistical conclusions on certain indicators of perceptions and practices juxtaposed with relevant demographic characteristics, a sample of the study population was taken. With an assumed 50% chance of having accepted EVD control interventions at a confidence interval of 95% with an error margin of 5%, a sample size of 384 was computed for the quantitative portion of the study for each area. Thus, for the two provinces, the sample size should total 768, which we rounded up to 800 to make allowance for losses. The size of the qualitative study was dependent on the saturation of information was collected from each category of respondents.

### Sampling strategy

A multi-stage sampling technique was adopted in selecting the communities, households, and respondents in this study. Two administrative areas—the epicenters of the EVD outbreak within each province—were purposively selected, and 10 communities were randomly selected from each of the two administrative areas.

Selection of the ***survey households*** and ***respondents***

The center of the selected community was the reference point on the map from which the team spun a pencil to determine the first route and first household; thereafter, we moved to the right to pick the next household, continuing until the necessary number of households for the sample was included. For neighborhoods with *cul-de-sacs*, the step was retraced, and a turn to the left and then to the right was made to continue the sampling process.

For each selected household, an adult (≥ 18 years) was randomly selected for inclusion as a participant in the study. We carefully alternated between sexes while selecting participants; if in household number one, a male was selected, in the next, we focused on selecting a female.

## Methods

The study was conducted with a mixed-methods approach using qualitative and quantitative techniques. The methodology for data gathering included in-depth interviews and focus group discussions as well as surveys using a structured questionnaire. These methods require a strong focus on individual actors rather than state actors^12^.

### Techniques of Data Collection

Focus group discussions (FGDs) and in-depth interviews (IDIs) were distributed among various community leaders and community members, as shown in Table 1 for North Kivu and the Ituri province.

We developed a set of questions covering different thematic areas to guide the discussions. The questions covered health care services in the community, awareness of and practices related to EVD, and assessments of the different pillars of the response interventions. For the FGDs, 8 to 12 people were selected for each session. A minimum of two FGDs were conducted in each of the selected communities. Separate FGD sessions were held for males and for females in each of the communities. Overall, eight FGD sessions were conducted in each province.

#### In-depth Interviews

The IDIs were conducted in each community wherein an FGD was conducted. The IDIs were held with community/ opinion leaders in the selected communities and the team leaders of response pillars. We used the interviews to explore people’s opinions, views, attitudes, practices, and insights regarding the outbreak, the corresponding response, and other socio-cultural factors that may have influenced their attitudes toward the response. The FGD guide was used for the in-depth interviews focusing on the thematic areas relevant to the evaluation.

### Survey Content and Data Collection

A structured questionnaire was used for collecting quantitative data from households. The questionnaire, which addressed all the indicators that were used for answering the research questions, was structured using results from the qualitative study. It was categorized into the following sections: socio-demographic data, perception of health problems in the community, knowledge of EVD, the perceived epidemiology of Ebola in the communities, and sources of EVD information. Others included issues on communication and community engagement, infection prevention and control in the communities, vaccination, and surveillance, as well as treatment and care. Other sets of questions covered safe and dignified burial, psychosocial, logistics, and security issues.

All interviews and discussions were tape-recorded, and detailed notes were taken simultaneously, including verbal citations. Tape-recorded interviews were transcribed according to standard rules. Observations were also recorded and, together with discussions and interviews, triangulated with the quantitative data to arrive at our conclusions.

### Training and Pilot Trials

All instruments were ***translated*** into Swahili and French, the common languages spoken in the communities, and back-translated to English for clarity of meaning. In each province, ***10 research assistants*** with substantial experience in interactive community research, the use of qualitative and quantitative techniques, and cultural sensibility were recruited and trained for three days in Beni and another three days in Bunia. The training covered the study objectives and use of the instrument for data collection. Training also included data entry into an ATLAS.ti template (qualitative data) and EPI INFO software (quantitative data). The instruments were reviewed after training to ensure clarity, understanding, and sensitivity. For each province, a ***supervisor*** worked with a principal investigator to monitor data quality, safety, and ethical research conduct, including in the management of informed consent procedures. The study was conducted first in Ituri, then in North Kivu. The lessons learned from Ituri were used to manage the process in the North Kivu province, which presented a more challenging environment in terms of security and logistics. The ***data analyst*** developed and pre-tested the template for data entry and analysis using the pilot test output. Considering the short study period, data were collected using pencil and paper instead of an android device. Fieldwork took 20 days to complete in each province before we could begin analysis and report writing.

### Data management

All **quantitative data** were double-checked by the researcher before being entered into the computer. Data were entered into EPI Info and processed using SPSS. Descriptive statistics were used to determine the proportions of various categories of respondents and indicators and for comparison. Frequency tables and graphic illustrations were used for presenting the data.

**Qualitative data** comprising FGDs and IDIS were transcribed from audio records to text. All textual data were analyzed using the Atlas.ti software package. Data were analyzed according to themes corresponding to the indicators in the quantitative data and triangulated during the presentation to enable complementary and analogous interpretation.

Considering the continuous analytical process involved in qualitative analysis, noteworthily, the initial analysis of the key informant interviews and focus group discussions informed the final development of the structured questionnaire to be used in the study. This further enhanced triangulation between the two sets of data to be collected. While the quantitative results provided us with statistical conclusions, the qualitative results emphasized what was actually said and provided illustrative quotes that contextualized and deepened the quantitative results.

### Ethical Considerations

The principle of do-no-harm was adhered to in the study. Informed study approval was obtained at the province, local administration, community, and household levels informed consent was obtained from all individuals that were involved in the study. The WHO/AFRO Ethics Review Committee provided ethical approval for the study. All researchers attended a mandatory training that included a substantial discussion of the related ethical research issues. Fifty percent of the research assistants were females, ensuring same-sex interviews and moderation of FGD sessions. The assistants were also trained and mandated to comply with child protection and gender sensitivity in the process of data collection and visits.

## Results and Discussion

This section presents the results of the interviews and focus groups, which we have grouped under three main themes.

### Perceptions and Representations of Diseases

The participants considered some diseases treatable by modern medicine, some as treatable exclusively with traditional medicine, and others as treatable by both types. Nevertheless, community members reported utilizing all of the healthcare options available to them in their environment. Examining the participants’ therapeutic itineraries reveals how, in numerous cases, they can be likened to “therapeutic roaming.” Further, by analyzing the responses regarding disease types that can be treated by traditional medicine, we determined that this method ranks high on the list of therapeutic choices and involves a wide field of available interventions that can be chosen based on perceptions and representations. In both North Kivu and Ituri, community members referred to certain diseases that they believe can only be effectively treated using traditional medicines (i.e., they believe modern/ orthodox medical treatments cannot be used to treat these conditions); in less than two months of fieldwork in North Kivu and Ituri, we were able to identify 75 such diseases (see Table 2).

In addition to the diseases specifically included in the questionnaire, 57 people mentioned other diseases, all of which are listed in Table 2. Some of the names given to these diseases are derived from responses grouped in the “other” category. In addition to the diseases mentioned in local languages, others were mentioned in other languages. For example, some respondents gave the disease names in French. These include hemorrhoids, hypertension, gastric syndromes, typhoid, diabetes, prostate disease, hernia, bone cancer, yellow fever, intestinal worms, poisoning, sexual dysfunction, epilepsy, leprosy, cancer, asthma, diarrhea, HIV/AIDS, and cough. Poisoning was cited under different names in native languages. Based on the analysis of the responses from the 57 respondents, some diseases were mentioned in combination with others. Therefore, there were more diseases mentioned than respondents, who replied differently from what had been presented and pre-coded in the questionnaire. Furthermore, some respondents mentioned more than one disease, and certain diseases were mentioned several times, such as poisoning. Table 1 does not present the frequency of a disease referred to more than once.

A disease is perceived in different ways based on its representations within the community. It is not always considered to have a mechanical cause from a known vector or agent but is often interpreted as the result of an evil deed, malevolent phenomenon, transgression, punishment, or the breach of a ban or taboo. An illness is, thus, perceived as the implementation of an action carried out by forces motivated by punishment and eventually manipulated by others. Thus, the search for healing not only involves a doctor or nurse but also someone with any knowledge regarding warding off bad luck and protecting against punishment or aggression. Healing involves neutralizing the source of evil and its cause; in this sense, the traditional healer becomes the most important person for treating a disease believed to be outside the realm of modern medicine. Several considerations can be drawn from these facts:

- Patients are referred to health facilities based on the specific disease diagnosed.
- For certain diseases, health facilities are not recommended.
- According to the interviews, poisoning or intoxication ranks as the first among these diseases; this rank was also confirmed by the quantitative survey.
- The use of modern medicine to treat these diseases would aggravate the patients’ conditions and lead to death.
- Certain diseases were considered incompatible with modern treatment.
- Responses suggested that any treatment’s effectiveness is due to the suitability of the treatment method for the specific disease.
- Diseases would respond to a given drug depending on disease origin.
- The body either accepts or rejects treatment depending on the perception or representation of the disease. Perceptions and representations constitute intangible factors of socio-cultural heritage, whereas what is known is part of cognitive heritage. However, in many cases, perceptions and representations are also considered “knowledge,” and they tend to replace cognitive heritage. Experiences and available resources play significant roles under this designation.
- The choice of treatment is not just a health issue but a cultural and societal issue.
- The traditional endogenous etiology and nosography become the *de facto* first criteria for therapeutic care choices.
- Death is the expected consequence of modern treatment for illnesses considered to be solely within the scope of traditional medicine.

During interviews with traditional healers and community leaders, one assertion was commonly made by both sides in various forms that can be summarized by what a traditional healer from Beni said:

“Some illnesses fall under the realm of traditional medicine. You waste your time trying to treat them with modern medicine. As soon as you use the wrong treatment for these types of illnesses, the patient inevitably dies. Many diseases do not accept such treatment.” According to some participants, a combined treatment would, in certain cases, be a death sentence.

Some diseases are within the scope of traditional medicine, while others can be treated by both fields of medicine. Perceptions and representations of these diseases are given more importance in traditional modern medicine. This perspective does not favor the use of health facilities and is against treating patients at Ebola treatment centers (ETCs). Some diseases are within the scope of traditional medicine.

**Figure 1:**
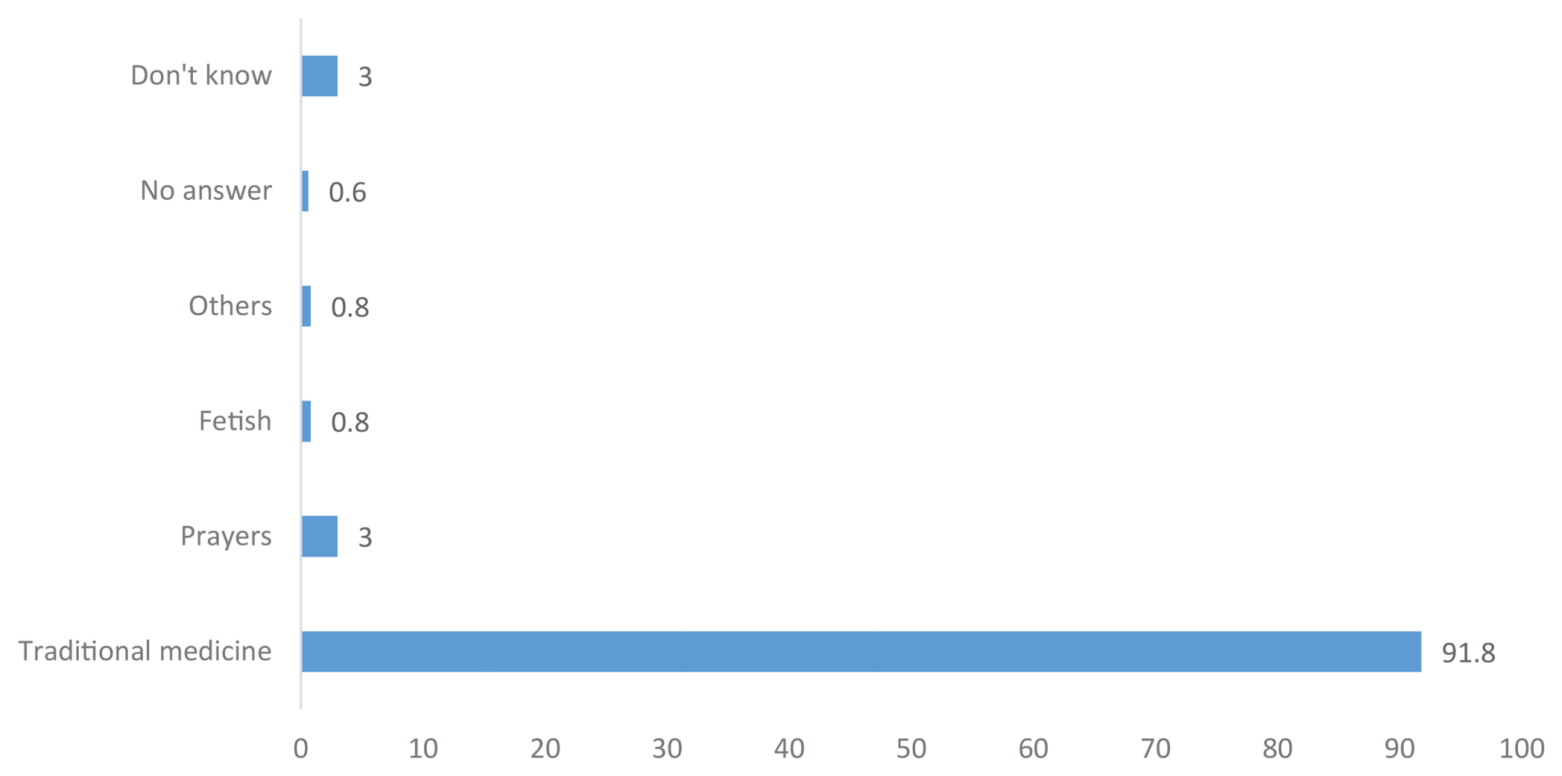
Perceived treatment for diseases that cannot be managed with orthodox medicine.

Traditional medicine is considered the appropriate remedy for most diseases that are not treatable by modern medicine (see Figure 1). However, prayers are also considered necessary, as is the use of a fetishist. In several cases, this first option is traditional medicine. In fact, among the traditional healers we interviewed, one was considered a fetishist. This respondent was the last resort after patients had tried everything else. The person who was supposed to accompany us to her house was reluctant at first; once we arrived, she left. However, this person was a traditional healer. Four people discussed other methods related to traditional medicine. It appears that traditional medicine is almost the exclusive option for illnesses not considered treatable by modern medicine.

**Figure 2:**
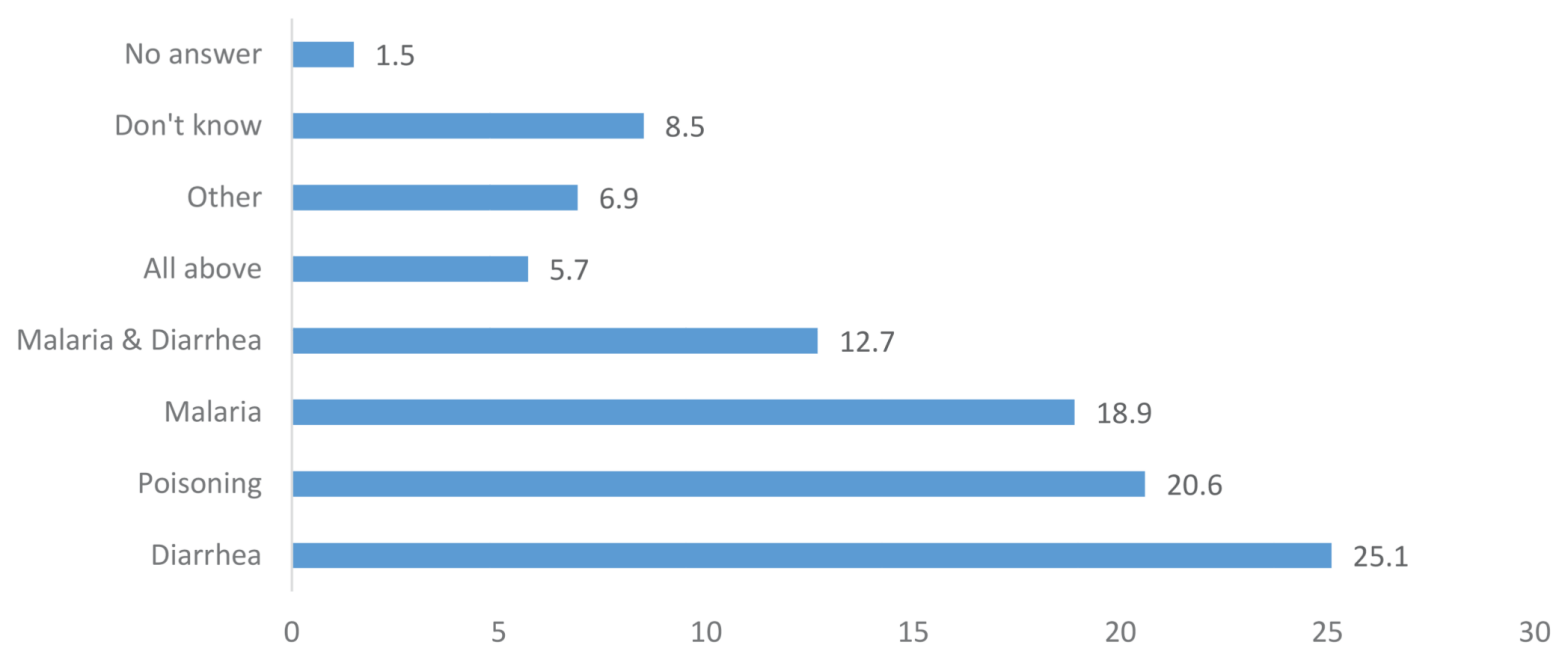
Disease with similar symptoms to EVD that cannot be treated by orthodox medicine.

According to Figure 2, diarrhea, malaria, and poisoning constituted 64.7% of the diseases mentioned as having the same symptoms as EVD. Some people mentioned both malaria and diarrhea, raising the total to 77.4%. Others said that all possibilities had the same symptoms as EVD. The most important message drawn from this data is that, for diseases that have the same symptoms as EVD, traditional medicine is also used. Thus, people who show signs of EVD are not referred to health centers as a priority. This leads to a delay in their treatment by the Ebola Response teams. This situation is likely to lead to EVD spread as the patient moves through the various possible treatment options of traditional medicine. There is also a process of delegitimization of modern medicine.

## Discussion

In this study, information about 75 diseases was provided by 57 of the 800 people surveyed using a questionnaire. These names were also mentioned in individual interviews with resource persons as well as in focus group discussions. For all diseases, possibilities for their treatment were also discussed. We classified these options into three groups: (i) diseases that are exclusively treated by traditional medicine, (ii) diseases that are exclusively treated by modern medicine, and (iii) diseases that are treated by both.

In this section, we also discuss treatment in ETCs, dignified and safe burials (DSBs), vaccination, and the rumors surrounding these activities. According to Table 2, modern medicine is not applied for over 70 diseases. Their names were given in the local languages spoken by the people we interviewed, including Kiswahili, Kinande, Lingala, Nour, Alur, and Kituba. In North Kivu and Ituri, traditional medicine is considered the only option to treat EVD, which is the case for 75 other diseases as well. Poisoning, which is among the diseases for which traditional medicine is used, is referred to under 21 names that vary from one community to the next. Most EVD survivors have sought to treat their illnesses as poisonings.

A disease can also occur as a consequence of a behavior. For example, participants described *bolombo* and *kabaku*, which are illnesses bestowed through an attack or spell, as forms of revenge, or as punishments for breaking a rule. Disease can also be a consequence of eating meat or another prohibited food. Thus, some diseases result from personal behaviors rather than natural occurrences. Further, a single disease can result from misbehavior such as aggression, attack, or revenge and can be caused by a spirit sent to bestow harm, such as the *mapepo*, a wind-traveling spirit.

By analyzing these diseases, we draw various conclusions, some of which are consistent with findings from a previous study of diseases in Equateur province^13^.

- **Disease as the consequence of behavior**. Some diseases are attributed to behavior. This is the case of ***libaku***, which refers to a disease caught in a field that affects the sole of the foot. It is caused by a spell cast on someone who steals crops from the field. It is not mere presence in the field but also wrongdoing that causes the disease.
- **Disease as the result of breaking a rule. *Libaku*** also fits within this framework. As stealing is not an approved behavior, one who does it suffers the consequences.
- **Disease as punishment. *Libaku*** is also a punishment for a behavior that is not valued.
- **Disease as a matter of personal responsibility**. In the case of ***bolombo***, the individual suffering from it has touched an unspecified prohibited object. Here, the behavior also causes the disease. We have also discussed the disease as an elementary form of behavior^14^.
- **Disease as the result of a transgression**. This is the case for ***libaku*** as well as ***bolombo***.
- **Disease with polyetiology. *Libaku*** and ***bolombo*** are examples of conditions with multiple etiologies. In some cases, the behavior is the cause of the disease; in other cases, the focus is centered more on the consequences of the behavior. However, personal responsibility is also involved. There is a causal chain of the disease and considering all the elements is important. This polyetiology can lead to treatment plurality and diverse therapeutic choices and therapeutic itineraries resulting in therapeutic roaming, as the various causes are not treated in the same way.
- **Aggression**. Irrespective of the cause, poisoning is considered to be present in all of these diseases, and it has different names that vary according to language, including ***awala, awola, Kahawa, karawa, kariwa, karfo, karlo, karoho, Karoo, karufo, Kisege, kisebue, lebako, lebaku, sumu, tcheche***, and ***tipo***.
- **Revenge**. Revenge is not directly expressed in the names of the diseases listed in Table 1. The causes leading to poisoning were not addressed, as they were not part of the aims of this research. Revenge is only discussed here as a hypothesis because it was not expressed by the participants or in words. This revenge is in the form of bewitchment or punishment^15^.
- **Disease coming from spirits**. The supernatural world manifests itself through diseases. This is the case with ***mapepo***.
- **Disease through consumption and diet**. The consumption of certain foods is responsible for certain diseases. For example, meat was mentioned as related to ***lugnama*** and ***regnama***.
- **The specificity of some diseases**. Some diseases are considered men’s diseases (dysfunction), women’s diseases (infertility, false pregnancy), and infant diseases (anal diseases).
- **Disease treatment**. For all the diseases mentioned, treatment is possible, even if traditional medicine is considered the only effective method.

The diversity mentioned by participants regarding the various diseases offers many possibilities for messages of awareness aiming to prevent the disease. For example, participants mentioned behavior (which includes conduct, actions, and compliance with preventive measures), contact, and dietary habits, especially regarding eating meat. It is, therefore, possible to use what was said about the diseases, even those considered treatable exclusively by traditional medicine, to discuss transmission and prevention of EVD. The existence of treatment is an opportunity to discuss EVD treatment within the exclusive domain of modern medicine. Moreover, it provides an opportunity to discuss the alternatives offered by some traditional healers, as discussed below. However, none of the traditional healer participants stated having the means to treat EVD. On the contrary, many of them have been involved in EVD *Response* activities. Additionally, there are local theories of the causes of misfortunes based on the representation of the body and disease^1^. For EVD, these theories are varied; they include sorcery attacks, theft, and eating meat, and have been discussed in previous studies^13^.

Communities perceive EVD as a mystery or witchcraft. Further, it is considered part of a conspiracy involving local, national, and international politicians^15^, which is consistent with findings from Equateur province^13^. The perception of risk varies according to the context. Additionally, when the disease is considered a divine punishment^16^, ETCs are not considered places for treatment. This aspect of punishment appears in the representations of the disease in Equateur as well as in North Kivu and Ituri^13, 17^. EVD representations exist in communities in other countries. For example, the concepts of *jok* and *gemo* can be spirits or gods that are not evil but rather providers of resources among the Acholi in Uganda and Gabon, but they can also be considered evil spirits. *Gemo* is seen as resulting from the lack of respect for honoring the gods^18^. This denomination is a cultic way to help understand the disease, and it underpins the practices associated with its treatment. There are other names for the disease in local languages that focus on trauma, isolation, stigmatization, and virulence of the disease, and some have variations that imply a relationship to gender^2^.

The representations surrounding EVD are often a source of fear for both patients and health personnel and even for decision-makers^15^. However, perceptions change as more information is shared and knowledge is gained about EVD^19^, as highlighted in a study of medical students at Yangon University. Representations of the extent and complexity of illnesses lead to a preference for traditional medicine^20^. However, often the perception of seriousness, if it leads to the use of traditional medicine, does not exclude the use of modern medicine^4^. Perceptions and representations of the modern healthcare system do not prohibit the use of self-medication and traditional medicine^4^.

However, the representations and perceptions of EVD favor the use of traditional medicine. These representations also concern EVD’s etiology and lead to certain therapeutic choices^21–23^. In particular, for any disease considered poisoning, traditional medicine is the preferred treatment option. Most of the population does not have a clear perception of EVD, as they have not experienced it^3^. Moreover, some perceive it as something evil, devilish, caused by witchcraft, or a curse, as mentioned above. EVD is also seen as a sign that the end of the world is approaching^3^.

Interviews with EVD survivors indicate that most of them (or their relatives) had considered their illness as poisoning. Even for some recovered healthcare workers, relatives had resorted to traditional treatment for poisoning. In these circumstances, while ETCs are the most appropriate place to treat EVD, the healthcare system under which they operate is not valued in this sense because of perceptions and representations of EVD within the communities in North Kivu and Ituri.

Perceptions and representations of diseases determine people’s treatment choices. The names given to diseases in North Kivu and Ituri communities help reveal their perceptions and representations. Over 70 names of diseases were collected from 57 people through a questionnaire survey, which was qualitatively analyzed. Three categories of diseases were identified: those treatable by modern medicine, those treatable by traditional medicine, and those treatable by both. Further, a disease is seen as more than an event; it is also connected to perceptions and representations regarding its etiology, and the etiology often determines the possibilities for its treatment. According to survey results, diseases can be the result of a curse, evil phenomenon, transgression, punishment, or breaking or disrespecting a prohibition or taboo. If it is a punishment, it originates from people’s actions that were meant to cause harm. Thus, a disease’s etiology reveals the source of the offending evil.

EVD is described as a devilish and demonic disease that stems from witchcraft. Traditional medicine is therefore considered the only way to treat it. In the studied provinces, the same is true for the 75 diseases that are considered untreatable by modern medicine. Poisoning was frequently mentioned among these diseases and is referred to by 21 different names in the communities. Some EVD survivors first treated their infection with EVD as a case of poisoning. Many diseases are considered to be the consequences of individual behaviors. For example, *bolombo* affects someone who has touched something that was forbidden. *Kabaku* is regarded as the result of a spell cast on someone who has stolen crops from a field, which constitutes a punishment for breaking a ban. In all these cases, the disease is related to personal responsibility. Personal responsibility is also involved in cases of illness arising from eating something specific and from revenge. However, certain diseases can also be construed as the consequences or results of aggression or attacks, as in poisoning cases. The attack can be made by sending a spirit with the intention to cause harm, as with *mapepo*, a spirit that travels through the wind. Sometimes, a single disease has several causes, such as aggression, attack, and revenge motivated by behaviors.

However, whatever the nature of a given disease, they are all considered treatable. The entire therapeutic mission involves finding the most appropriate form of care: traditional medicine, modern medicine, or both. In some cases, however, the simultaneous use of both approaches is avoided, as it could aggravate certain diseases. The use of combined treatments, for example, could lead to death depending on the illness. In fact, the participants of this study indicated that the compatibility between the disease and treatment type often ensures the effectiveness of the latter. However, most of our questionnaire respondents considered some diseases to be treatable only through traditional medicine (this situation was also observed by Diarra^17^ in the Equateur province in 2018, and many of these illnesses share symptoms with EVD. Therefore, many of those who have recovered from EVD began their treatment with traditional medicine. In some cases, however, the simultaneous use of both modern and traditional medicine is considered incompatible; the mixed approach is believed to aggravate certain diseases and even sometimes lead to death.

Therefore, matching the disease to the appropriate type of treatment is crucial for effectiveness. The body becomes a site for treatments that can be accepted or rejected according to the perception or representation of the disease. Traditional etiology and nosography, as they appear in the endogenous perceptions and representations of the disease, become the primary criteria for therapeutic choices.

## Conclusion

In the communities in the provinces of North Kivu and Ituri in the Democratic Republic of the Congo, in general, the perceptions and representations of diseases do not promote the use of modern medicine. This is particularly an obstacle to certain activities related to the *Response* to the EVD epidemic, such as the use of treatment centers.

The ways in which diseases are perceived reveal their etiology, which, in turn, indicates the possible forms of treatment. Disease perception and representation are used to divide diseases and their treatments into three distinct categories: (i) diseases that can be treated exclusively by traditional medicine, (ii) diseases that can be treated exclusively by modern medicine, and (iii) diseases that can be treated by both. Additionally, several causes are typically attributed to a single disease; for example, a disease could occur as a punishment or as the consequence of a curse, evil phenomenon, transgression, or broken taboo. EVD is described as a devilish or demonic disease stemming from witchcraft or a conjuring attack. In North Kivu and Ituri, traditional medicine is not only considered the best option for treating EVD but also 75 other diseases mentioned by research participants. One of these, poisoning, was referred to by 21 different terms that varied from one community to the next. Most individuals who have recovered from EVD had been previously treated for poisoning.

Diseases can have several causes and are often related to personal behavior and responsibility. ***Bolombo***, for example, is an attack on someone who has touched something forbidden. ***Kabaku*** comes from a spell cast on someone who has stolen crops from a field. Similarly, EVD is the manifestation of a form of punishment and the sanction for breaking a ban. The same is true of a disease that results after eating something. Poisoning is also thought to derive from an aggression or attack and can be caused by a spirit sent to do harm, for example, a *mapepo*, a wind-traveling spirit. Whether coming from aggression or revenge, the disease reflects a person’s behavior. Nevertheless, a single disease can be caused by various elements, such as aggression, attack, and revenge motivated by behavior.

All diseases are considered treatable, and it is assumed the cure is simply a matter of determining the most appropriate remedy, either with traditional or modern medicine or both. However, most participants believed some diseases could only be treated with traditional medicine, many of which show the same symptoms as EVD. While both modern and traditional medicine can be used simultaneously in treatment, for some diseases, the use of both methods can aggravate the infection and result in a fatal outcome.

## Figures and Tables

**Table 1:** Distribution of participants in the IDI and FGD sessions by provinces

Target	North Kivu				Ituri Province			
	Butembo		Beni		Mbuti		Bunia	
	IDI	FGD	IDI	FGD	IDI	FGD	IDI	FGD
Pillar leads	All		All		All		All	
Pillar members	2/pillar		2/pillar		2/pillar		2/pillar	
Community leaders^1^	≥2/ community		≥2/ community		≥2/ community		≥2/ community	
Leader of survivor group	≥2/ community		≥2/ community		≥2/ community		≥2/community	
Community adult males		≥2 groups		≥2 groups		≥2 groups		≥2 groups
Community: adult females		≥2 groups		≥2 groups		≥2 groups		≥2 groups
Community male youth		≥2 groups		≥2 groups		≥2 groups		≥2 groups
Community female youth		≥2 groups		≥2 groups		≥2 groups		≥2 groups
Survivors		≥2 groups		≥2 groups		≥2 groups		≥2 groups

1 These include traditional, religious, political and social opinion leaders

**Table 2:** Diseases considered treatable only by traditional medicine.

No.	Disease name	Community in which the disease was mentioned		Disease description by community members
		North Kivu	Ituri	
31	Karuwa Kinande		X	Poisoning
32	Katche	X	X	Intestinal worms
33	Kateke Kibila		X	Infant disease
34	Kawgolo		X	Intoxication causing a dry cough
35	Kichwakulu Kibila		X	Turtle disease
36	Kichwa Kabambi	X		Headaches
37	Kicuo Kabangi Kiswahili	X	X	Macrocephalus
38	Kifoko Kibila		X	Not specified
39	Kifubo	X		Stomachal (gastric)and anal wound
40	Kisebue		X	Poisoning
41	Kisege Alur, Kilindu		X	Poisoning
42	Kifobo Kibila		X	Lower limb paralysis in children
43	Kifubo		X	Diarrhea diseases in newborns
44	Kichwakulu	X	X	Transformation of the skin
45	Koriwa		X	Poisoning
46	Kumugongo Kibila	X	X	Infant anal wound
47	Kwab		X	Not specified
48	Kwao		X	Poisoning
49	Lebako/Lebaku		X	Poisoning
50	Libaku		X	Foot disease, a spell cast by a thief
51	Likwonga		X	Itching
52	Lugnama		X	Illness caused by eating meat
53	Mapasu		X	Not specified
54	Mapepo Kiswahili		X	Evil spirits
55	Malange	X		Paralysis, bones are affected
56	Mahuha			Asthma
57	Musipa Yakulum Kiswahili		X	Nerve pain, epilepsy
58	Matori		X	Jaundice, eye disease
59	Moyasay		X	Not specified
60	Mutuo munene Lingala			Macrocephalic
61	Mwnamimbi	X		Female infertility
62	Nyoka ya batoto		X	Intestinal worms in children
63	Nyoka ya kyanaume	X		Infertility in men
64	Ratoya		X	Poisoning causing skin scabies
65	Regnama Kibila		X	Disease due to eating smoked meat
66	Rutowa		X	Poisoning
67	Suluba Kiswahili		X	Measles
68	Sumu Kiswahili	X	X	Poisoning
69	Tcheche Kilindo		X	Poisoning
70	Tipo		X	Evil spirit from a family member
71	Tipo Alur		X	Poison
72	Tsokombera Tsokombela		X	Not specified
73	Tutubanga	X		Swollen belly in women
74	Wula		X	Infant disease
75	Vukerere	X		Hip disease
